# Correlations between choroidal thickness and renal function in patients with retinal vein occlusion

**DOI:** 10.1038/s41598-020-74058-0

**Published:** 2020-10-08

**Authors:** Sang Uk Choi, Ja Young Oh, Jee Taek Kim

**Affiliations:** Department of Ophthalmology, College of Medicine, Chung-Ang University Hospital, 102 Heukseok-ro, Dongjak-gu, Seoul, 06974 South Korea

**Keywords:** Nephrology, Predictive markers, Retina, Imaging and sensing

## Abstract

The purpose of this study was to analyze the correlation of renal function indices with sub-foveal choroidal thickness (SFChT) in treatment-naïve (naïve) eyes with retinal vein occlusion (RVO) using swept-source optical coherence tomography (SS-OCT) and systemic workup. Retrospective chart review was performed from Dec 2016 to Sep 2019 in patients newly diagnosed with treatment-naïve unilateral RVO. Ocular parameters, including SFChT, and systemic profiles, including renal function indices, were reviewed. Simple and multiple linear regression analyses were performed to check if there was a correlation between renal profiles and SFChT. A total of 56 patients were included in the study; 34 of them were branch RVO and 22 were central RVO patients. Multiple linear regression analysis revealed that SFChT was positively correlated with estimated glomerular filtration rate (eGFR) (*P* < 0.001). SFChT showed significant correlation with renal function indices. In addition, choroidal thickness may be considered as systemic biomarkers for renal function.

## Introduction

Retinal vein occlusion (RVO) is a common retinal disease in adults and has a prevalence of 0.52%–0.77%^1,2^. It is a major cause of visual impairment due to macular edema, retinal ischemia, and neovascular complications^3^. Epidemic studies have revealed that hypertension (HTN) is the strongest risk factor for RVO^1,4^. Moreover, RVO is a systemically important disorder because it itself is associated with increased risk of cardiovascular disease^5,6^.

Optical coherence tomography (OCT) is a useful noninvasive imaging tool for the in vivo visualization of the chorioretinal microstructure^7^. With recent technical developments, enhanced depth imaging OCT and swept-source OCT (SS-OCT) can visualize the deep choroid layer with accurate segmentation and high clarity^8^. Using these techniques, several studies have reported changes in sub-foveal choroidal thickness (SFChT) in eyes with RVO^8,9^. However, they have reported inconsistent findings. Du et al. reported that SFChT was significantly thinner in the RVO group than in the control group^8^. Tang et al. reported that SFChT was significantly thicker in eyes with RVO than in fellow eyes^9^. These inconsistent reports of SFChT in eyes with RVO suggest that it is affected by some confounding factors^8,9^. Moreover, Balmforth et al. reported that choroidal thickness is associated with renal function in patients with chronic kidney disease (CKD)^10^. Furthermore, Mule et al. also reported a correlation of SFChT with renal function in patients with HTN^11,12^.

RVO and CKD share a major independent risk factor, HTN^1,13^. Interestingly, the renal podocyte, which can be damaged by high blood pressure, has very similar microstructure and function as the vascular pericyte^14^. Thus, we hypothesized impaired kidney function caused by HTN to have a close correlation with SFChT in patients with RVO. Therefore, we analyzed the correlation of renal parameters with SFChT in patients with RVO.

## Results

### Baseline characteristics

A total of 415 patients with RVO visited the retina clinic, of whom the 56 patients (branched RVO, n = 34; central RVO, n = 22) who met the inclusion criteria and not the exclusion criteria were included in the present study. The mean age of the patients was 58.8 ± 11.9 years (range 18–85 years), mean body mass index (BMI) was 25.5 ± 3.5 kg/m^2^, and mean onset time for RVO was 32.4 ± 35.9 days (range 1–90 days). Fifty two out of 56 patients had hypertension with well-controlled blood pressure using medications. Six patients had hyperlipidemia. The mean arterial pressure was 93.2 ± 13.5 mmHg (81.1–108.3 mmHg), and the mean duration of hypertension was 6.8 ± 8.4 years (range, 0.5–30 years).

### Comparison between RVO eyes and normal fellow eyes

Central retinal thickness (RT) and mean RT were significantly higher in eyes with RVO than in normal fellow eyes (*P* < 0.001; *P* < 0.001). SFChT was significantly higher in eyes with RVO (316.1 ± 101.3 μm) than in normal fellow eyes (290.8 ± 100.5 μm) (*P* = 0.015). The mean choroidal thickness (ChT) was also significantly higher in eyes with RVO (285.6 ± 86.2 μm) than in normal fellow eyes (269.2 ± 59.4 μm) (*P* = 0.014). The interobserver reproducibility of ChT ranged from 0.986 to 0.993. Ocular parameters in eyes with RVO and normal fellow eyes are compared in Table 1.

**Table 1:** Ocular parameters of eyes with retinal vein occlusion and fellow eyes.

Values	Eyes with RVO (n = 56)	Fellow eye (n = 56)	*p* value*
BCVA, Log MAR (range)	0.38 ± 0.56 (0–1.6)	0.05 ± 0.23 (0–1)	0.002
Intraocular pressure, mmHg (range)	15.1 ± 2.4 (10–20)	15.5 ± 3.6 (11–20)	0.551
Central retinal thickness, μm (range)	423.9 ± 200.1 (203–624)	233.0 ± 18.1 (204–263)	< 0.001
Mean retinal thickness, μm (range)	391.3 ± 109.9 (254.6–535.2)	283.5 ± 17.4 (240.56–746.67)	< 0.001
Subfoveal ChT, μm (range)	316.1 ± 101.3 (132–616)	290.8 ± 100.5 (119–573)	0.015
Mean ChT, μm (range)	285.6 ± 86.2 (117–555)	269.2 ± 59.4 (99.86–379)	0.014

*BCVA* best-corrected visual acuity, *ChT* choroidal thickness.

*Paired t-test.

### Comparison between RVO eyes and normal control eyes

Eyes with RVO were compared with eyes in age-matched normal control group (n = 56). Age, BMI, and IOP were not significantly different between the two groups. Central and mean RT were also significantly higher in eyes with RVO than in normal control eyes (*P* < 0.001; *P* < 0.001). However, SFChT and mean ChT were not significantly different between eyes with RVO and normal control eyes (*P* = 0.167; *P* = 0.280). The comparison is shown in Table 2.

**Table 2:** Comparison between eyes with retinal vein occlusion and normal control eyes.

	Eyes with RVO (n = 56)	Normal subject (n = 56)	*p* value*
Age, years (range)	58.8 ± 11.9 (29–85)	60.6 ± 14.2 (38–76)	0.598
BMI, kg/m^2^ (range)	25.5 ± 3.5 (19.04–34.93)	25.2 ± 2.9 (20.69–31.29)	0.770
BCVA, Log MAR (range)	0.38 ± 0.56 (0–1.6)	0.05 ± 0.08 (0–0.3)	< 0.001
Intraocular pressure, mmHg (range)	15.1 ± 2.4 (10–20)	15.89 ± 3.2 (10–21)	0.640
Central retinal thickness, µm (range)	423.9 ± 200.1 (203–624)	222.6 ± 22.8 (202–276)	< 0.001
Mean retinal thickness, µm (range)	391.3 ± 109.9 (254.6–535.2)	278.4 ± 13.0 (255–307)	< 0.001
Subfoveal ChT, µm (range)	316.1 ± 101.3 (132–616)	298.7 ± 15.3 (268–322)	0.167
Mean ChT, µm (range)	285.6 ± 86.2 (117–555)	279.2 ± 16.9 (239–292)	0.280

*BCVA* best-corrected visual acuity, *ChT* choroidal thickness.

*Independent t-test.

### Simple and multiple linear regression analysis

**Figure 1:**
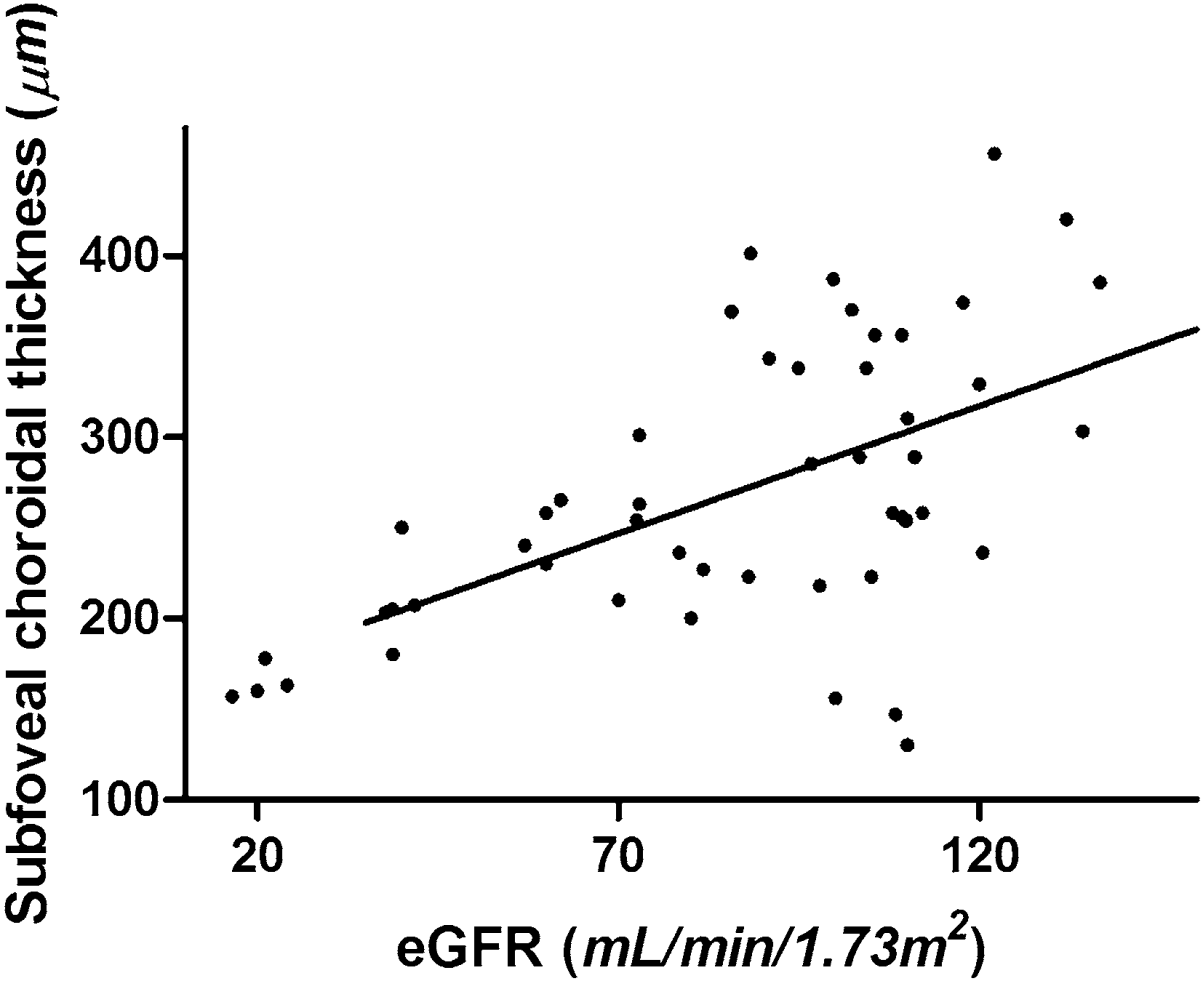
Scatter plot with positive correlation between subfoveal choroidal thickness and estimated glomerular filtration rate.

In simple linear regression analysis, SFChT of normal fellow eyes was significantly associated with BMI (*P* = 0.028), age (*P* = 0.003), serum osmolarity (*P* = 0.035), creatinine (*P* = 0.002), estimated GFR (estimated glomerular filtration rate) (*P* < 0.001), and phosphorus levels (*P* = 0.016). Stepwise multiple linear regression revealed that SFChT had a significant positive correlation with eGFR (*P* < 0.001; Table 3, Fig. 1).

**Table 3:** Simple and multiple linear regression analyses of the association between subfoveal choroidal thickness and systemic profiles in fellow eyes of retinal vein occlusion.

Parameters	Single linear regression			Multiple linear regression	
	β*	R^2^	*p* value	β*	*p* value^‡^
BCVA, Log MAR	52.81	0.025	0.343		
Intraocular pressure, mmHg	− 0.962	0.002	0.79		
Refraction error, diopter	2.462	0.001	0.834		
BMI, kg/m^2^	− 7.863	0.124	0.028	− 0.149	0.341
Mean AP, mmHg	− 0.651	0.013	0.484		
Age, years	− 1.968	0.09	0.003	− 0.243	0.061
HTN duration, years	− 3.494	0.074	0.125		
Glucose	− 0.01	− 0.028	0.952		
Serum osmolarity	− 0.344	0.094	0.035	− 0.273	0.468
Total cholesterol	0.006	0.04	0.694		
BUN, mg/dL	− 1.957	0.031	0.285		
Creatinine, mg/dL	− 91.270	0.232	0.002	− 0.185	0.542
BUN/Cr ratio	1.563	0.037	0.243		
eGFR, mL/min/1.73 m^2^	1.393	0.301	< 0.001	1.391	< 0.001
Phosphorus, mg/dL	− 45.505	0.148	0.016	− 0.103	0.621
Calcium, mg/dL	− 7.342	0.002	0.772		

*Standardized (β) coefficient.

^†^Stepwise multiple univariate linear regression analysis; R^2^ = 0.30.

## Discussion

In this study, we evaluated the correlation of renal function with choroidal thickness using SS-OCT. This study revealed that choroid thickness was closely correlated with eGFR in patients with RVO, independently of age and other potentially confounding factors.

The correlation of choroidal thickness with renal function has been rarely described with reports only by few studies^10–12^. Balmforth et al. reported that chorioretinal thinning in patients with CKD is associated with lower eGFR and greater proteinuria^10^. Mule et al. described a correlation between SFChT and eGFR in patients with HTN^11,12^. In patients with retinal disease, however, a close correlation has not been identified, even though patients with retinal disease frequently suffer from systemic diseases such as HTN or CKD. Moreover, RVO is a representative disease that is affected by the systemic condition. Thus, we investigated the systemic effect on choroidal thickness in eyes with RVO. Furthermore, there have been conflicting results for choroidal thickness in eyes with RVO. Du et al. reported that SFChT was significantly thinner in the RVO group than in the control group^8^. Tang et al. reported that SFChT was significantly thicker in eyes with RVO than in fellow eyes^9^. These inconsistencies in SFChT in eyes with RVO suggest that it is affected by some confounding factors^8,9^. Thus, these are the reasons why we have tried to reveal the effects of systemic factors in patients with RVO.

BRVO and CRVO are different diseases. However, they have similar phenotypes, similar pathophysiology, and common cardiovascular risk factors^15,16^. The primary purpose of this study is to reveal the association between choroidal thickness in normal contralateral eyes and systemic states because macular edema and RVO affect choroidal thickness. Thus, we considered BRVO and CRVO under the same category. CRVO usually involves the macula, whereas BRVO may not always involve the macula. Therefore, macular involvement by RVO may affect choroidal thickness. In this study, however, there were no BRVO patients in whom the macula was not involved.

Elevated arterial pressure is a major risk factor of both RVO and kidney damage^3,13,17^. The glomerulus is the target structure of hypertensive kidney damage due to its extensive vascular network^18^. The choroid is also a highly vascularized tissue and is very similar to the glomerulus in terms of anatomical structure and physiological function^19,20^. Moreover, both vascular structures are affected by the regulation of localized and systemic renin–angiotensin–aldosterone hormonal cascades^14,21^. The anatomic and physiologic similarity of the tissues may lead to simultaneous damage.

The retina has been considered an important organ for non-invasive evaluation of microvasculature through the diagnosis of diabetic or hypertensive retinopathy^7,22,23^. Our findings suggest that SFChT may be used as a potential biomarker for subclinical or clinical renal dysfunction, even though the parameters would require adjustment for age, axial length, and macular edema.

In addition, we found that SFChT was greater in eyes with RVO than in normal fellow eyes. Several studies reported that the choroid was significantly thicker in eyes with RVO or the occlusive area than in contralateral eyes or the non-occlusive area^9,24–26^. Our results were consistent with these studies. It is known that that the choroid is regulated by autonomic and sensory innervation^27^. Thus, the first possible explanation for thicker choroid in RVO eyes is that local autoregulatory myogenic mechanism (associated with vascular smooth muscles) caused by ischemic retina leads to compensatory increase in choroidal blood flow and consequently to choroidal thickening^27^. The second possible explanation is that the choroid becomes edematous passively by the intravitreal elevation of the vascular endothelial growth factor (VEGF) level. Several studies showed that choroidal thickness in eyes with RVO decreased after anti-VEGF injection^24,28^. From these previous studies, we can hypothesize that the choroid tissue is sensitive to intravitreal VEGF concentration. Accordingly, in this study, we used contralateral eyes without RVO to investigate if there is a correlation between SFChT and renal function. However, in a report by Du et al., SFChT was significantly thinner in the group with RVO than in the group without RVO in a large population-based study^8^. We additionally compared eyes with RVO and normal control eyes. SFChT was greater in eyes with RVO than in normal control eyes. However, the difference was not significant. In addition, SFChT was lesser in contralateral eyes than in normal control eyes. However, the difference was not significant here either. The systemic status of RVO patients may act as a confounding factor and lead to a large standard deviation in SFChT in patients with RVO. Consequently, this may lead to no significant statistics. Thus, in this study, when compared with contralateral eyes, eyes with RVO had a thicker choroid. However, eyes with RVO did not have a thicker choroid than normal control eyes. Interestingly, this discrepancy is similar to the previous conflicting results^8,9^. Given the effect of renal function on the choroid, the SFChT of eyes with RVO should be compared with that of contralateral eyes without RVO.

Additionally, Rayess et al. reported baseline SFChT as a predictor of improvement in visual acuity after anti-VEGF injection in both BRVO and CRVO^29,30^. The baseline SFChT of functional responder (BCVA gain ≥ 2 lines) was greater than that of nonresponders in BRVO and CRVO^29,30^. Although Rayess et al. did not consider renal function, we carefully suggest that functional recovery in patients with RVO is associated with renal function through the results of the present study.

This study has several limitations. First, this was a retrospective observational study. Thus, the timing of OCT was not controlled (diurnal variations were not considered). Second, this study has relatively few patients because systemic evaluation was not routinely performed in patients with RVO. Third, only patients whose systemic workup was performed within a span of four weeks from initial retinal evaluation were included. This interval could affect the systemic status. Fourth, this study did not investigate the relationship between functional outcome and renal dysfunction because of the cross-sectional nature of this study. Fifth, axial length was not considered although myopic and hyperopic eyes were excluded. Finally, retinal hemorrhage and edema may have affected the measurements of SFChT of eyes with RVO. However, multiple regression analysis for our key findings used normal contralateral eyes. A prospective future study should provide stronger evidence for correlation between SFChT and renal dysfunction.

In conclusion, SFChT was positively correlated with eGFR. It is the first glance to reveal the association between choroidal thickness and kidney function in treatment-naïve RVO patients.

## Methods

### Study design

This retrospective cross-sectional study was approved by the institutional review board committee of Chung-Ang University Hospital in Seoul, South Korea (IRB #1701-004-16029) and adhered to the tenets of the Declaration of Helsinki. Informed consent was waived by the institutional review board committee of Chung-Ang University Hospital in Seoul because of the retrospective nature of the study.

### Study subjects

The medical records of newly diagnosed patients with treatment-naïve unilateral RVO (macular involving) at Chung-Ang University Hospital between Dec 1, 2016, and Sep 30, 2019, were retrospectively reviewed. Among them, only patients whose renal function was evaluated within a span of 4 weeks from retinal evaluation were selected. The following parameters were recorded from medical charts: age, gender, mean arterial pressure (mmHg), BMI (km/m^2^), HTN duration, serum level of glucose, serum osmolarity [(2 × (sodium + potassium)) + (blood urea nitrogen (BUN)/2.8) + (glucose/18)], and serum level of total cholesterol.

In addition, the following renal parameters were recorded from laboratory examination: BUN, creatinine, BUN-to-creatinine ratio (BUN/Cr), eGFR, phosphorus, and calcium. The exclusion criteria were as follows: other chorioretinal disorders including drusen, age-related macular degeneration, diabetic retinopathy, eyes with treatment history using laser and intravitreal injection, myopic or hyperopic eyes with refractive error >  ± 3.0 D, and eyes with low-quality OCT images due to media opacity (i.e., low image quality index < 90). In addition, patients with a history of smoking, glaucoma, ocular trauma, ocular inflammation, ocular ischemic syndrome, or any type of intraocular surgery (except cataract surgery) were excluded as well. The patients with systemic disease including history of angina, acute myocardial infarction, acute coronary syndrome or coronary revascularization (with a stent or coronary artery bypass graft), or significant plaque on coronary angiography, acute or uncontrolled hypertension (systolic BP ≥ 150 mmHg, diastolic BP ≥ 90 mmHg), significant carotid artery stenosis, history of a carotid endarterectomy were excluded as well.

### Ophthalmic examination and diagnosis of retinal vein occlusion

Patients had undergone a comprehensive ophthalmic evaluation, including slit-lamp biomicroscope examination, fundus examination, fundus photographs, and SS-OCT. The measurement of best-corrected visual acuity, refractive error, and intraocular pressure was also done. The determination of RVO presence was based on retinal examination, photographs, and SS-OCT.

### Optical coherence tomography

**Figure 2:**
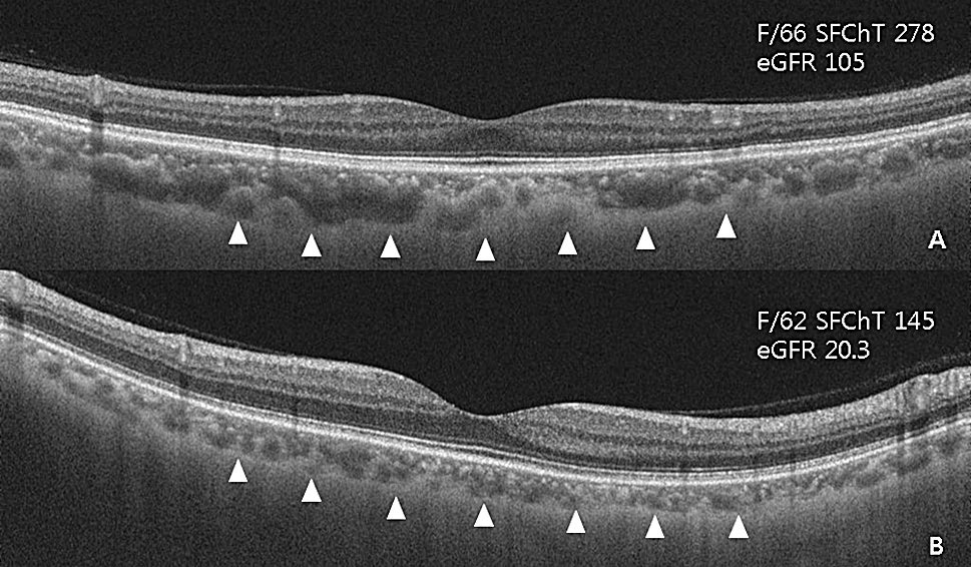
Representative swept-source optical coherence tomography image of a contralateral normal eye in patients with retinal vein occlusion. A. 66-year-old female patient with retinal vein occlusion. Estimated glomerular filtration rate (eGFR) was 105 mL/min/1.73 m^2^; subfoveal choroidal thickness was 278 μm. B. 62-year-old female patient with retinal vein occlusion. Estimated glomerular filtration rate (eGFR) was 20.3 mL/min/1.73 m^2^; subfoveal choroidal thickness was 145 μm. Arrow heads indicate chorioscleral interface.

OCT imaging was done using Deep Range Imaging (DRI)-OCT Triton (Topcon, Tokyo, Japan). The device provided a 7 μm axial and 20-μm horizontal resolution and a scan speed of 100,000 A-scans per second using a swept wavelength light source. Central RT and ChT were defined and measured using the built-in software as previously described (Fig. 2)^31^. ChT was measured additionally at 1000-μm intervals from the fovea to 3000 μm to nasal and temporal area.

### Statistical analysis

Two independent masked observers (OJY and JTK) measured ChT, and the averaged values were used for statistical analyses. Data are presented as means ± standard deviations. Statistical analyses were performed using SPSS version 25.0 software (IBM Corp., Armonk, NY, USA). The baseline characteristics of patients were compared with those of normal subjects using independent t-tests. Ocular parameters of eyes with RVO were compared with those of normal fellow eyes (without RVO) using paired t-test. The correlations of SFChT with renal profile were analyzed using simple and multiple linear regression analyses. *P* values < 0.05 were considered statistically significant.

## Data Availability

The datasets during and/or analysed during the current study are available from the corresponding author on reasonable request.

## References

[1] Song P, Xu Y, Zha M, Zhang Y, Rudan I (2019). Global epidemiology of retinal vein occlusion: a systematic review and meta-analysis of prevalence, incidence, and risk factors. J. Glob. Health.

[2] Rogers S (2010). The prevalence of retinal vein occlusion: pooled data from population studies from the United States, Europe, Asia, and Australia. Ophthalmology.

[3] Jonas JB, Mones J, Glacet-Bernard A, Coscas G (2017). Retinal vein occlusions. Dev. Ophthalmol..

[4] Wang S (2009). Major eye diseases and risk factors associated with systemic hypertension in an adult Chinese population: the Beijing Eye Study. Ophthalmology.

[5] Wong TY (2005). Cardiovascular risk factors for retinal vein occlusion and arteriolar emboli: the Atherosclerosis Risk in Communities & Cardiovascular Health studies. Ophthalmology.

[6] Cugati S (2007). Retinal vein occlusion and vascular mortality: pooled data analysis of 2 population-based cohorts. Ophthalmology.

[7] Mule G, Vadala M, Geraci G, Cottone S (2018). Retinal vascular imaging in cardiovascular medicine: new tools for an old examination. Atherosclerosis.

[8] Du KF (2013). Subfoveal choroidal thickness in retinal vein occlusion. Ophthalmology.

[9] Tang F (2019). Comparison of subfoveal choroidal thickness in eyes with CRVO and BRVO. BMC Ophthalmol..

[10] Balmforth C (2016). Chorioretinal thinning in chronic kidney disease links to inflammation and endothelial dysfunction. JCI Insight.

[11] Vadala M (2019). Retinal and choroidal vasculature changes associated with chronic kidney disease. Graefes Arch. Clin. Exp. Ophthalmol..

[12] Mule G (2019). Association between early-stage chronic kidney disease and reduced choroidal thickness in essential hypertensive patients. Hypertens. Res..

[13] Haroun MK (2003). Risk factors for chronic kidney disease: a prospective study of 23,534 men and women in Washington County, Maryland. J. Am. Soc. Nephrol..

[14] Wong CW, Wong TY, Cheng CY, Sabanayagam C (2014). Kidney and eye diseases: common risk factors, etiological mechanisms, and pathways. Kidney Int..

[15] Zhou JQ (2013). The 10-year incidence and risk factors of retinal vein occlusion: the Beijing eye study. Ophthalmology.

[16] Cugati S, Wang JJ, Rochtchina E, Mitchell P (2006). Ten-year incidence of retinal vein occlusion in an older population: the Blue Mountains Eye Study. Arch. Ophthalmol..

[17] Ponto KA (2015). Prevalence and risk factors of retinal vein occlusion: the Gutenberg Health Study. J. Thromb. Haemost..

[18] Breyer MD, Susztak K (2016). The next generation of therapeutics for chronic kidney disease. Nat. Rev. Drug. Discov..

[19] Izzedine H, Bodaghi B, Launay-Vacher V, Deray G (2003). Eye and kidney: from clinical findings to genetic explanations. J. Am. Soc. Nephrol..

[20] Zipfel PF, Heinen S, Jozsi M, Skerka C (2006). Complement and diseases: defective alternative pathway control results in kidney and eye diseases. Mol. Immunol..

[21] Wilkinson-Berka JL, Agrotis A, Deliyanti D (2012). The retinal renin-angiotensin system: roles of angiotensin II and aldosterone. Peptides.

[22] Cheung CY, Ikram MK, Sabanayagam C, Wong TY (2012). Retinal microvasculature as a model to study the manifestations of hypertension. Hypertension.

[23] Wong TY (2004). Retinal microvascular abnormalities and renal dysfunction: the atherosclerosis risk in communities study. J. Am. Soc. Nephrol..

[24] Kim KH (2015). Regional choroidal thickness changes in branch retinal vein occlusion with macular edema. Ophthalmologica.

[25] Okamoto M, Yamashita M, Sakamoto T, Ogata N (2018). Choroidal blood flow and thickness as predictors for response to anti-vascular endothelial growth factor therapy in macular edema secondary to branch retinal vein occlusion. Retina.

[26] Esen E, Sizmaz S, Demircan N (2016). Choroidal thickness changes after intravitreal dexamethasone implant injection for the treatment of macular edema due to retinal vein occlusion. Retina.

[27] Reiner A, Fitzgerald MEC, Del Mar N, Li C (2018). Neural control of choroidal blood flow. Prog. Retin. Eye Res..

[28] Tsuiki E, Suzuma K, Ueki R, Maekawa Y, Kitaoka T (2013). Enhanced depth imaging optical coherence tomography of the choroid in central retinal vein occlusion. Am. J. Ophthalmol..

[29] Rayess N (2019). Baseline choroidal thickness as a short-term predictor of visual acuity improvement following antivascular endothelial growth factor therapy in branch retinal vein occlusion. Br. J. Ophthalmol..

[30] Rayess N (2016). Baseline choroidal thickness as a predictor for treatment outcomes in central retinal vein occlusion. Am. J. Ophthalmol..

[31] Kim JT, Park N (2020). Changes in choroidal vascular parameters following pan-retinal photocoagulation using swept-source optical coherence tomography. Graefes Arch. Clin. Exp. Ophthalmol..

